# T-wave morphology abnormalities in the STREAM stage 1 trial

**DOI:** 10.1080/14740338.2024.2322116

**Published:** 2024-03-10

**Authors:** Gareth Hughes, William J. Young, Henry Bern, Angela Crook, Pier D. Lambiase, Ruth L. Goodall, Andrew J. Nunn, Sarah K. Meredith

**Affiliations:** a MRC Clinical Trials Unit at UCL, Institute of Clinical Trials and Methodology, London, UK; b Centre for Clinical Pharmacology and Precision Medicine, William Harvey Research Institute, Queen Mary University of London, London, UK; c Barts Heart Centre, St Bartholomews Hospital, Barts Health NHS Trust, London, UK; d Institute of Cardiovascular Science, University College London, London, UK; e NIHR Barts Biomedical Research Centre, London, UK

**Keywords:** Rifampicin-resistant tuberculosis, moxifloxacin, clofazimine, T wave morphology, QTcF, electrocardiogram

## Abstract

**Background:**

Shorter regimens for drug-resistant tuberculosis (DR-TB) have non-inferior efficacy compared with longer regimens, but QT prolongation is a concern. T-wave morphology abnormalities may be a predictor of QT prolongation.

**Research design and methods:**

STREAM Stage 1 was a randomized controlled trial in rifampicin-resistant TB, comparing short and long regimens. All participants had regular ECGs. QT/QTcF prolongation (≥500 ms or increase in ≥60 ms from baseline) was more common on the short regimen which contained high-dose moxifloxacin and clofazimine. Blinded ECGs were selected from the baseline, early (weeks 1–4), and late (weeks 12–36) time points. T-wave morphology was categorized as normal or abnormal (notched, asymmetric, flat-wave, flat peak, or broad). Differences between groups were assessed using Chi-Square tests (paired/unpaired, as appropriate).

**Results:**

Two-hundred participants with available ECGs at relevant times were analyzed (QT prolongation group *n* = 82; non-prolongation group *n* = 118). At baseline, 23% (45/200) of participants displayed abnormal T-waves, increasing to 45% (90/200, *p* < 0.001) at the late time point. Abnormalities were more common in participants allocated the Short regimen (75/117, 64%) than the Long (14/38, 36.8%, *p* = 0.003); these occurred prior to QT/QTcF ≥500 ms in 53% of the participants (Long 2/5; Short 14/25).

**Conclusions:**

T-wave abnormalities may help identify patients at risk of QT prolongation on DR-TB treatment.

**Trial Registration:**

The trial is registered at ClinicalTrials.gov (CT.gov identifier: NCT02409290). Current Controlled Trial number, ISRCTN78372190

## 1. Introduction

The STREAM Stage 1 trial of treatment for rifampicin-resistant tuberculosis demonstrated the non-inferiority of a Short 9-month regimen compared to the contemporaneous WHO-recommended 20+ month Long regimen [1]. From a safety perspective, a higher proportion of participants on the Short regimen (which included high-dose moxifloxacin and clofazimine – Table 1) experienced clinically relevant QT prolongation (defined as QT/QTcF ≥500 ms or ≥60 ms increase from baseline) compared to the long regimen (which included standard-dose fluoroquinolone and no clofazimine) although the difference was not significant. QT prolongation can have serious implications for patient safety due to an increased risk of malignant ventricular arrhythmia such as Torsades de Pointes, which can lead to sudden cardiac death; there were no cases in the current study. The primary mechanism of QT prolongation associated with fluoroquinolones, clofazimine, and many other drugs, is through blockade of the human ether-a-go-go-related-gene (*hERG*) potassium channels, which leads to prolongation of cardiac repolarization [2,3]. The diagnosis of acquired Long QT Syndrome (a-LQTS) caused by drug toxicity usually relies on measurement of the QT interval alone. In contrast, the diagnosis of congenital LQTS (c-LQTS) utilizes both QT interval measurements and specific T-wave morphology abnormalities including T-wave alternans and notching, which can be indicative of abnormal ventricular repolarization. It is recognized that some patients with c-LQTS and pathogenic variants, have normal QT intervals but an abnormal T-wave morphology [4,5], making assessment of T-wave abnormalities a useful diagnostic tool to prevent development of life-threatening arrhythmias.

**Table 1.** Summary of short regimen for STREAM stage 1.

			Weight group	
Phase and duration	Drug	Less than 33 kg	33 kg to 50 kg	More than 50 kg
Intensive and continuation (40 weeks)	Moxifloxacin	400 mg	600 mg	800 mg
	Clofazimine	50 mg	100 mg	100 mg
	Ethambutol	800 mg	800 mg	1200 mg
	Pyrazinamide	1000 mg	1500 mg	2000 mg
Intensive (16 weeks)	Isoniazid	300 mg	400 mg	600 mg
	Prothionamide	250 mg	500 mg	750 mg
	Kanamycin*	15 mg per kilogramme (kg)body weight (maximum 1 g)		

*Kanamycin given three times weekly after 12 weeks.

There are several T-wave morphology abnormalities such as notching, asymmetry, and flatness that can be used as biomarkers for *hERG* potassium channel blockade [6–10]. A recently published large review of 270, 039 individuals investigated T-wave morphology and risk of mortality [11]. The study demonstrated a higher adjusted mortality hazard ratio for patients with higher morphology combination scores (MCS) made up of asymmetric, flat, and notched T-waves compared to those with lower MCS values and showed T-wave morphology can provide important prognostic information independent of other factors including QT interval. In a separate cohort study of 23,962 low to moderate risk participants, an association was found between T-wave morphologic variations and life-threatening ventricular arrythmias that was independent of corrected QT interval [12].

Morphology combination scores are often used to assess T-wave abnormalities via software such as QT Guard Plus (GE Healthcare); however, these require digitalized ECGs which were not available from STREAM Stage 1. These are also not available on the majority of ECG machines used in clinical practice, particularly in low- and middle-income countries treating a disproportionately large number of multi-drug resistant TB (MDR-TB) patients worldwide. Several studies have relied on visual inspection of T-wave morphology to identify abnormalities, which may be more practical in programmatic MDR-TB settings [13–17].

The WHO 2020 MDR-TB guidelines grouped drugs from A to C in decreasing order of preference. Four of the seven drugs in the first two groups are known to prolong the QT interval (moxifloxacin, levofloxacin, bedaquiline, and clofazimine); however, less is understood about their effect on T-wave morphology. Some studies have suggested T-wave asymmetry, notching, and flatness with moxifloxacin use [8,17], although there have been inconsistencies in the literature [18]. Whether T-wave morphology abnormalities can be used as a predictive tool in people being treated for MDR-TB is unknown.

ECGs from STREAM Stage 1 provide a unique data set to investigate the effects of drugs known to prolong the QT interval, which were taken for 9 months on the short regimen and 20 months on the long regimen, in a geographically diverse population.

These exploratory analyses aimed to address the following objectives:

1. To determine whether T-wave morphology abnormalities occurred before or after the development of clinically relevant QT prolongation.
2. To assess whether the frequency of T-wave morphology abnormalities consistent with *hERG* potassium channel disruption differed between regimen and risk group.
3. To assess whether T-wave morphology abnormalities occurred more frequently at the late time point (12–36 weeks) compared with the early (1–4 weeks) or baseline readings.

## 2. Patients and methods

STREAM Stage 1 had 424 randomized participants, allocated in a 2:1 ratio to either the Short, 9- month regimen (282 participants), or the long regimen taken for 20 or more months (142 participants). Treatment could be extended if participants were smear positive at the end of the intensive phase. All participants had regular ECG monitoring to week 52 following randomization although some participants did not have ECG readings available for all time points due to missed visits, withdrawal from the study or death. The process for ECG review was modified over the course of the trial. Whilst the QT and QTc intervals from machine readings were recorded by participating sites for all trial participants and sent to the coordinating center, the ECG tracing was not always sent for every visit. A baseline ECG was available for 411 (97%) of 424 randomized patients, and at least one early ECG (weeks 1–4) for 81% and one late time point ECG (weeks 12–36) for 72% of the participants.

The selection criteria for this study included participants with an available ECG at each of the time-points (baseline, early and late). All participants from either regimen who developed clinically relevant QT prolongation (≥500 ms or increase of ≥60 ms from baseline) with available ECGs were then selected and referred to as ‘high-risk’ (*n* = 82). A random sample of 118 out of 191 ‘low-risk’ participants with ECGs available and without QT prolongation was selected, representative of the study population in respect to country, gender, and age. This number was deemed sufficient to answer the study objectives.

There were an additional 26 high-risk participants who were excluded from this study as they had missing ECGs.

**Figure 1.**
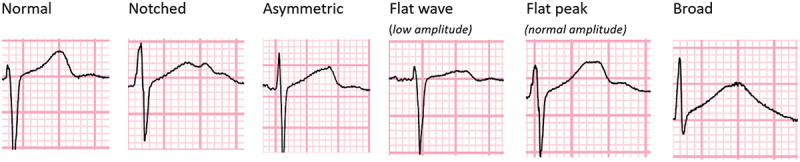
Examples of the different T-wave morphology that were categorized from participants in the STREAM stage 1 trial.

Following a period of training and validation with a cardiologist, an infectious diseases physician experienced in the management of DR-TB patients reviewed all 12 leads for each of the ECGs and categorized them into normal, notched, asymmetric, flat-wave (<1 mm amplitude of T wave peak), flat-peak (>1 mm amplitude of T wave peak) and broad (width ≥2 × height of T wave) (Figure 1). The same abnormal categories present in two or more adjacent leads were counted.

The review of ECGs was carried out blind to the allocated regimen, participant ID, time point, and automated QT interval reading. For leads with mixed T-wave morphology, the categorization was based on the most frequent morphology observed. The analyses presented are based on the abnormalities which occurred in any of the 12 leads and did not look at specific regions, e.g. inferior leads to assess whether certain abnormalities occurred more frequently there compared to anterior leads.

We analyzed the frequency of abnormalities between regimen and risk groups using chi-square tests. Logistic regression, with standard errors adjusted to account for the repeated measurements (multiple ECG readings within a person), was used for the analysis of T-wave abnormalities over time. Both analyses were done in STATA version 17.0. Objective 1 was a descriptive analysis.

The Ethics Advisory Group of the International Union Against Tuberculosis and Lung Disease (The Union, and its North American affiliate, Vital Strategies, the trial sponsor), and all relevant national and local ethics committees approved the trial. Informed consent was sought for the original study. This study describes secondary analyses of trial data; further informed consent was not sought. This research was conducted in with the Helsinki Declaration as revised in 2013.

## 3. Results

In total, 600 ECGs were reviewed, one at each of the 3 time points for 200 participants. These comprised 118 participants in the low-risk group (59% of the total) and 82 high-risk (41%); 58 participants (29%) had been randomized to the long regimen, and 142 (71%) to the short regimen (Table 2). Of the 82 high-risk participants (74 short, 8 long regimen), 30 (36.6%) developed QT or QTcF prolongation ≥500 ms (25 short regimen: 5 long regimen) and 78 (95.1%) (72 short regimen: 6 long regimen) developed a ≥60 ms increase in their QTcF from baseline. Some of the 82 high-risk participants met both criteria for QT prolongation.

**Table 2.** Summary of the 200 participants by regimen and risk group.

	Short regimen (*n* = 142)	Long regimen (*n* = 58)
High risk (*n* = 82)	74	8
Low risk (*n* = 118)	68	50

A summary of T-wave morphology for all 600 ECGs is provided in Table 3. Almost, a third of ECGs (195; 32.5%) had evidence of at least one of the five abnormalities. Some ECGs displayed more than one abnormal category so the total number when individual categories are combined, exceeds 195. At baseline, 36 participants had one abnormality, 1 had two abnormalities and none had three or more abnormalities. On treatment, 78 participants had one abnormality, 20 had two abnormalities and 3 had three or more abnormalities.

**Table 3.** Summary of T-wave morphology categories for all 600 ECGs reviewed.

	Normal	Any abnormality	Notched	Asymmetric	Flat-wave	Flat peak	Broad
N (%)	405 (67.5)	195 (32.5)	29 (4.8)	73 (12.2)	70 (11.6)	19 (3.2)	42 (7)

### 3.1. Timing of T-wave morphology abnormality in relation to development of clinically relevant QT prolongation

In total, 77% (23/30) of participants with contemporaneous or subsequent QT/QTcF ≥500 ms had evidence of T-wave morphology abnormalities (Table 4). We found 10% (3/30) of participants had T-wave morphology abnormalities after having already reached a QT/QTcF ≥500 ms. Few participants [13% (4/30)] developed a QT/QTcF ≥500 ms with no evidence of the relevant T-wave abnormalities in the ECGs that were reviewed. A similar pattern was seen in those who developed a QTcF ≥60 ms above baseline with 63% (49/78) of participants showing evidence of T-wave morphology abnormalities before or at the same time as having reached the QTcF threshold.

**Table 4.** Timing of T-wave morphology abnormality in relation to development of QT/QTcF prolongation.

Timing	Participants	%
**QT/QTcF ≥500 ms**	**Total participants (*n* = 30)**	
Abnormal before QT/QTcF >500	16	53.3
Abnormal same time QT/QTcF >500	7	23.3
Abnormal after QT/QTcF >500	3	10.0
No abnormality	4	13.3
**QTcF ≥60 ms above baseline**	**Total participants (*n* = 78)**	
Abnormal before QTcF ≥60 ms	31	39.7
Abnormal same time QTcF ≥60 ms	18	23.1
Abnormal after QTcF ≥60 ms	12	15.4
No abnormality	17	21.8

### 3.2. Frequency of T-wave morphology abnormality by regimen

A comparison of emerging abnormalities between participants randomized to each regimen is shown in Table 5; this analysis excludes baseline ECGs and ECGs while on treatment from participants with that abnormality at baseline. More participants on the Short regimen had at least one emerging ECG abnormality compared to the Long regimen; 64% (75/117) vs 36.8% (14/38), *p* = 0.003.

**Table 5.** Comparison of T-wave abnormalities in regimen groups.

	Regimen		
	Long	Short	P-value
Any abnormality	*N* = 38	*N* = 117	0.003
No	24 (63.2%)	42 (36%)	
Yes	14 (36.8%)	75 (64%)	
Notched	*N* = 56	*N* = 141	0.317
No	51 (91.1%)	121 (85.2%)	
Yes	5 (8.9%)	20 (14.8%)	
Asymmetric	*N* = 47	*N* = 130	
No	42 [89.4%]	99 [76%]	
Yes	5 [10.6%]	31 [24%]	**0.054**
Flat wave	*N* = 52	*N* = 130	
No	46 [88.4%]	104 [80%]	
Yes	6 [11.5%]	26 [20%]	0.176
Flat peak	*N* = 58	*N* = 142	
No	57 [98.3%]	124 [87.3%]	
Yes	1 [1.7%]	18 [12.7%]	**0.017**
Broad	*N* = 56	*N* = 140	
No	50 [89.3%]	112 [80%]	
Yes	6 [10.7%]	28 (20%]	0.121

Data shown are number of participants based on two ECGs per participant (percentage of participants).

Analysis for each individual abnormality showed a statistically significant difference between regimens for asymmetric and flat peak T-waves.

### 3.3. Frequency of T-wave morphology abnormality by risk group

A significant difference was also observed between risk groups with 72% (46/64), *p* = 0.002 of high-risk participants having demonstrated T-wave abnormalities versus 47% (43/91) low-risk participants (Table 6). The high-risk group also had significantly more notched and broad T waves than the low-risk group.

**Table 6.** Comparison of T-wave abnormalities by risk groups.

	Risk		
	Low	High	P-value
Any abnormality	*N* = 91	*N* = 64	0.002
No	48 [52.7] (%)	18 (28%)	
Yes	43 [47.3] (%)	46 (72%)	
Notched	*N* = 115	*N* = 81	**0.013**
No	106 (92.2%)	65 (80%)	
Yes	9 (7.8%)	16 (20%)	
Asymmetric	*N* = 100	*N* = 77	
No	83 [83%]	58 [75%]	
Yes	17 [17%]	19 [25%]	0.209
Flat wave	*N* = 112	*N* = 70	
No	97 [86.6%]	53[75.7%]	
Yes	15 [13.4%]	17 [24.3%]	0.06
Flat peak	*N* = 118	*N* = 82	
No	106 [90%]	75 [91%]	
Yes	12 [10%]	7 [9%]	0.698
Broad	*N* = 116	*N* = 80	
No	102 [88%]	60 [75%]	
Yes	14 [12%]	20 (25%]	**0.019**

Data shown are number of participants (percentage of participants).

### 3.4. Frequency of T-wave morphology abnormalities across time

**Figure 2.**
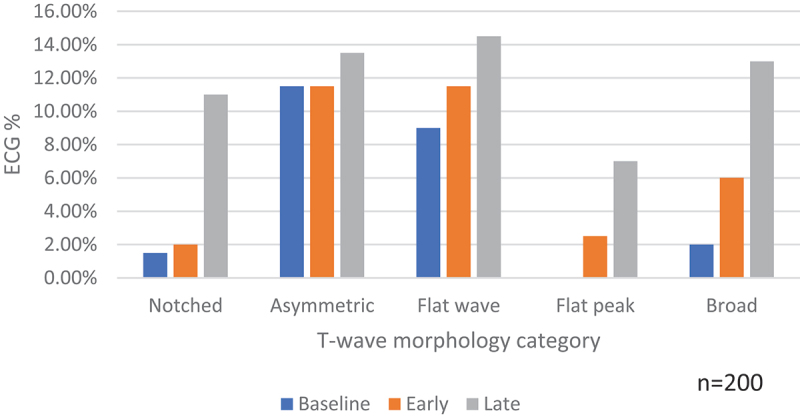
T-wave morphology abnormality by time point showing percentage of ECGs affected.

An increase in the frequency of abnormalities over time was observed. At baseline 22.5% (45/200) of participants had at least one of the five abnormalities present in ≥2 adjacent leads in the same ECG, compared to 30% (60/200) at the early time point and 45% (90/200), *p* < 0.001 at the late time point (Table 7, Figure 2). This pattern was observed in all categories except for asymmetric T-waves.

**Table 7.** Summary of T-wave morphology abnormalities at each of the three time-points.

	ECG time point			
	Base (*n* = 200)	Early (*n* = 200)	Late (*n* = 200)	P value
Normal	155 (77.5%)	140 (70.0%)	110 (55.0%)	**<0.001**
Any abnormal	45 (22.5%)	60 (30.0%)	90 (45.0%)	
Normal or non-notched abnormality	197 (98.5%)	196 (98.0%)	178 (89.0%)	**<0.001**
Notched	3 (1.5%)	4 (2.0%)	22 (11.0%)	
Normal or non-asymmetric abnormality	177 (88.5%)	177 (88.5%)	173 (86.5%)	0.541
Asymmetric	23 (11.5%)	23 (11.5%)	27 (13.5%)	
Normal or non-flat-wave abnormality	182 (91.0%)	177 (88.5%)	171 (85.5%)	0.087
Flat-wave	18 (9.0%)	23 (11.5%)	29 (14.5%)	
Normal or non-flat peak abnormality	200 (100.0%)	195 (97.5%)	186 (93.0%)	**<0.001**
Flat peak	0 (0.0%)	5 (2.5%)	14 (7.0%)	
Normal or non-broad abnormality	196 (98.0%)	188 (94.0%)	174 (87.0%)	**<0.001**
Broad	4 (2.0%)	12 (6.0%)	26 (13.0%)	

In contrast to Table 7, which showed the total number of patients with T-wave abnormalities at each time point, Table 8 summarizes the number of patients across the three time points to show which abnormality was present and whether the same patient displayed that abnormality at more than one time point. The first column indicates whether any of the five abnormalities were present rather than the same type which is shown in the remaining columns.

**Table 8.** T-wave abnormalities present across the three time points.

	Any	Notched	Asymmetric	Flat-wave	Flat peak	Broad
Type	Count (*n* = 134)	Count (*n* = 28)	Count (*n* = 59)	Count (*n* = 50)	Count (*n* = 19)	Count (*n* = 38)
Baseline only	14 (10.4%)	2 (7.1%)	12 (20.3%)	5 (10.0%)	0 (0.0%)	3 (7.9%)
Early only	26 (19.4%)	4 (14.3%)	14 (23.7%)	13 (26.0%)	5 (26.3%)	9 (23.7%)
Late only	48 (35.8%)	21 (75.0%)	20 (33.9%)	16 (32.0%)	14 (73.7%)	22 (57.9%)
Baseline and early	4 (3.0%)	0 (0.0%)	6 (10.2%)	3 (6.0%)	0 (0.0%)	0 (0.0%)
Baseline and late	12 (9.0%)	1 (3.6%)	4 (6.8%)	6 (12.0%)	0 (0.0%)	1 (2.6%)
Early and late	15 (11.2%)	0 (0.0%)	2 (3.4%)	3 (6.0%)	0 (0.0%)	3 (7.9%)
All timepoints	15 (11.2%)	0 (0.0%)	1 (1.7%)	4 (8.0%)	0 (0.0%)	0 (0.0%)

Most participants (66%, 88/134) had evidence of an abnormality on at least one reading but only 23% (31/134) of participants had abnormalities across two time points and 11% (15/134) across all three. The highest frequency of individual abnormalities was at the late time point ECG.

## 4. Discussion

This study found that T-wave morphology abnormalities consistent with *hERG* blockade occurred in a trial population that received DR-TB treatment and were present in a third of ECGs reviewed. These abnormalities occurred in a larger proportion of participants randomized to the short regimen as well as those who developed clinically relevant QT prolongation. They were more common at the late time point compared to baseline (before treatment had started) or the early time-point (within a month of treatment initiation). T-wave abnormalities also occurred prior to the development of QT prolongation in a large proportion of participants. Identification of T-wave morphology abnormalities could be used for risk stratification and to guide clinicians as to which patients need closer monitoring.

The association between certain T-wave morphology abnormalities (e.g. notched, asymmetric, and flat) is well established in c-LQTS [9,19,20]. Some of these abnormalities have also been observed in patients with coronary artery disease and electrolyte deficiencies but are less well established in those with a-LQTS [7,14]. To the best of our knowledge, these abnormalities have not been demonstrated before in a population being treated for MDR-TB which requires treatment for at least 6–9 months.

The abnormal T-wave morphology described in this study may allow clinicians to identify which patients are most at risk of QT prolongation and sudden cardiac death and undertake closer monitoring. An ECG at baseline could be an opportunity to assess T-wave morphology as well as the QT interval. We found that T-wave morphology abnormalities occurred early in follow-up, prior to the development of QT prolongation. Over half had evidence of the abnormalities before they reached a QT/QTcF ≥500 ms. This may allow identification of patients at risk, early in their treatment course at which point increased monitoring or adjustments to their regimen could be made before they reach a point where they are at increased risk of cardiac arrythmia. As the number of high-risk participants on the long regimen was small in comparison to the short regimen, analyses between regimens were limited as well as the fact that the analyses presented elsewhere has suggested the short regimen is more of an issue than the long.

TB disproportionately affects low- and middle-income countries. ECGs are noninvasive, inexpensive, and widely available, and are therefore a useful investigation to monitor patient safety and establish arrhythmic risk. In addition, as fluoroquinolones and clofazimine are also used in other settings such as *Mycobacterium abscessus, Mycobacterium leprae,* and complex bone and joint infections, these findings may also have broader relevance.

Early and late time points were chosen as early abnormalities in T-wave morphology may be more useful as a predictive tool and given the pharmacokinetic properties of moxifloxacin with a T_max_ of 0.75–3.5 hours, it may be possible to see T-wave morphology abnormalities as early as week 1. Late time points from week 12 allowed investigation of the impact of clofazimine on T-wave morphology as it has a long half-life (approximately 25–34 days), reaching a steady plasma state after 18–21 weeks. Some ECGs at baseline had an abnormal T-wave morphology that could indicate an underlying susceptibility to *hERG* blockade, or subclinical cardiovascular disease such as coronary disease, which will further increase their risk of clinically relevant QT interval prolongation.

Differences existed in the frequency of some of the individual types of morphology abnormality between groups. For example, notched and broad T-waves occurred significantly more frequently in high-risk participants than low-risk but not in short regimen versus long regimen participants. This suggests these specific T-wave morphological abnormalities may be more sensitive for identifying patients at risk and could indicate an underlying susceptibility for QT prolongation. The asymmetric and flat-peak T-waves were significantly more common in the short regimen but not in high-risk participants, suggesting this could be an effect related to the high dose fluoroquinolone in combination with clofazimine that does not necessarily translate to prolongation of ventricular repolarization time. These findings would benefit from further work to explore these differences.

The analyses of abnormalities by time point suggested that they seemed to evolve on treatment; it may be that the longer patients were exposed to the drugs the greater the disruption of their voltage gated ion channels. Unfortunately, ECGs after treatment completion were not available for all participants. Future work should investigate whether T-wave abnormalities resolve after treatment completes and how quickly this occurs.

### 4.1. Guidance for clinicians from this work

This study has shown T-wave morphology abnormalities may be a useful tool in monitoring cardiac safety for patients on MDR-TB treatment. Many clinicians involved in their management may be unaware of the significance of the T-wave abnormalities described e.g. notched, flat, asymmetric.

We have demonstrated that a clinician experienced in the management of TB with no cardiology background can be trained (with around 3 sessions of 15 hours in total) to identify the T-wave morphology abnormalities described using a simple visual manual method. Though it is recognized that this may not be practical for the many nurses who manage patients in high-burden countries and therefore QT/QTc measurements should remain the most important cardiac safety indicator, T-wave morphology could complement this.

Advanced computer programs utilizing machine learning can now be applied to detect subclinical Long QT syndrome in gene carriers and these could be implemented in a population such as this to predict QT prolongation or warn of T-wave changes to highlight the risk of this occurring [21]. Indeed, machine learning predicted QT prolongation and Torades de Pointes in a study of the *hERG* channel blocker Sotalol and has recently been reported [22].

Once T-wave abnormalities are identified, clinicians could increase the frequency of patient monitoring and check for electrolyte (potassium, magnesium, calcium) deficiencies and thyroid function which may exacerbate the problem. If these are normal, the abnormalities persist and QT prolongation worsens, treatment modification is likely to be needed. This should initially be interruption of the relevant drugs followed by dose reduction and lastly regimen change if there is no resolution. Early liaison with a cardiologist for review of ECGs and advice would also be advisable.

### 4.2. Limitations

This study had limitations.

1. Machine readings of the QT interval were used to decide high- and low-risk participants. As manual calculation is more accurate, it is possible there may have been fewer high-risk participants and more low-risk participants in these analyses.
2. The categorization of T-wave morphology relied on visual interpretation rather than computer software like QT Guard Plus (GE Healthcare). However, this could also be considered a strength as many doctors in low-middle-income countries in programmatic settings would also likely rely on visual interpretation rather than advanced computer programs.
3. Some high-risk patients did not have evidence of the T-wave morphology categories described, though not all ECGs on treatment were reviewed. Only one from the early time point and one from the late time point were chosen. It is possible that there were more morphology abnormalities that were not detected due to only two ECGs on treatment being reviewed per participant. Some low-risk participants had T-wave abnormalities, though to a lesser extent than the high-risk group.
4. Information on treatment changes and other factors that could have affected the morphology such as electrolyte deficiencies were not included in the analysis.
5. ECG leads with none of the specific five abnormalities described earlier were categorized as ‘normal’ rather than abnormal that included those of poor quality that were unreadable (<10% of ECG leads affected).
6. We were unable to analyze post-treatment ECGs on everyone. If we could have demonstrated T-wave morphology abnormalities reduced after completion of treatment this would have strengthened the case for concluding that these are treatment associated changes.
7. This was a post-hoc analysis of ECGs collected for the STREAM Stage 1 trial.
8. Just under half of the low-risk group had abnormal T-wave morphology. Although the specificity of 53% is not ideal, the sensitivity of 72% shows these abnormalities were present in nearly three-quarters of those who developed clinically relevant QT prolongation.

### 4.3. Strengths

This study had several strengths.

1. The participants included were a mix from both regimens and included those we know developed clinically relevant QT prolongation and those we know who did not. A number of different ethnic groups were included, and the low-risk group were a random representation of the study population with respect to age, gender, and site.
2. A large number of participants and ECGs were reviewed, just under half of the entire trial population.
3. ECGs were picked from three separate time points. This allowed identification of preexisting abnormalities at baseline, development of new abnormalities at later time points and the effect of time on treatment.
4. Prior to the review and categorization of ECGs, a period of training took place with a cardiologist specializing in electrophysiology disorders which included independent assessments of 30 ECGs followed by a comparison of categorizations to provide a quality check.
5. All the ECGs reviewed were blinded to study number, machine QT interval, regimen, and risk group. This reduced the chance of bias and confounding and adds strength to the validity of the findings.

## 5. Conclusion

This study has shown for the first time that patients on MDR-TB therapy develop T-wave abnormalities specific to pathology in the voltage gated potassium channels of the myocardium. The analyses also showed clear evidence that the abnormalities occurred prior to the development of clinically relevant QT prolongation, which may be useful for monitoring purposes. Significantly more T-wave abnormalities occurred in participants on the short regimen (with high-dose moxifloxacin and clofazimine) versus the long regimen (with standard dose fluoroquinolone only) and those that developed clinically relevant QT prolongation (high-risk) versus those that did not (low-risk). Finally, a significantly higher proportion of T-wave abnormalities occurred at the late time point compared to baseline.
